# Implementation Experiences of a School-Based Adolescent Nutrition Education Intervention: A Post-Intervention Qualitative Study Guided by Consolidated Framework for Implementation Research and the Social Ecological Model

**DOI:** 10.3390/nu18213652

**Published:** 2026-11-03

**Authors:** Humaira Mahmood, Babar Tasneem Shaikh, Farrah Pervaiz, Azka Naseem, Mehvish Idrees, Abdul Momin Rizwan Ahmad, Yasir Waheed, Saleh A. Alsanie

**Affiliations:** 1 School of Public Health, Health Services Academy, Islamabad 44000, Pakistan; humaira.talha.7@gmail.com (H.M.); shaikh.babar@gmail.com (B.T.S.); 2 Department of Community Medicine, Rawalpindi Medical University, Rawalpindi 46000, Pakistan; farrahpervaiz@gmail.com; 3 Department of Research and Development, Rawalpindi Medical University, Rawalpindi 46000, Pakistan; dr.azkanaseem@gmail.com; 4 Public Health Department, University of Azad Jammu and Kashmir, Muzaffarabad 13100, Pakistan; drmehvishidrees@gmail.com; 5 Department of Human Nutrition and Dietetics, NUST School of Health Sciences, National University of Sciences and Technology (NUST), Sector H-12, Islamabad 44000, Pakistan; 6 Department of Health Sciences, University of York, York YO10 5DD, UK; 7 NUST School of Health Sciences, National University of Sciences and Technology (NUST), Sector H-12, Islamabad 44000, Pakistan; yasir.waheed@nshs.nust.edu.pk; 8 Irfan Suat Gunsel Operational Research Institute, Near East University, 99138 Nicosia, Turkey; 9 Faculty of Health Sciences, Széchenyi István University, Egyetem Square 1, 9026 Győr, Hungary; 10 Department of Health Sciences, VIZJA University, 01-043 Warsaw, Poland; 11 Department of Basic Health Sciences, College of Applied Medical Sciences, Qassim University, Buraydah 52581, Saudi Arabia; 12 Department of Clinical Nutrition, Medical City, Qassim University, Buraydah 52581, Saudi Arabia

**Keywords:** adolescent nutrition, school-based intervention, nutrition education, implementation science, social ecological model, consolidated framework for implementation research (CFIR), Pakistan

## Abstract

Background/Objectives: School-based nutrition education provides a platform that could help in the promotion of healthy eating habits among adolescents. Nonetheless, information about the factors influencing the success of these interventions in low- and middle- income countries is still lacking. The objective of the study was to identify facilitators and barriers to effective implementation of school-based nutrition education by applying the Consolidated Framework for Implementation Research (CFIR) and interpreting findings across levels of the Social Ecological Model (SEM). Methods: A qualitative study was conducted after eight to ten weeks of nutrition education intervention in an urban school setting. Data were collected through two focus group discussions with sixteen students (eight students in each group), in-depth interviews with four teachers, six parents and key informant interviews with two school administrators. A semi-structured, CFIR- and SEM-based guide was used to conduct the data collection. Thematic analysis was conducted following Braun and Clarke’s six-phase approach, with deductive and inductive coding conducted independently by two researchers and reconciled through consensus. Results: Five themes were aligned with CFIR domains, including intervention characteristics, outer setting, inner setting, character of individuals, and implementation process. In general, the stakeholders agreed to the importance of relevance and contextualization of the intervention and the use of multi-mediums (classroom-based and online) to deliver the intervention, which they perceived to support improved knowledge of nutrition among adolescents. Implementation of the programme faced challenges arising from the incongruence between messages delivered in classrooms and those served in the school canteens, economic hurdles from home, cultural practices related to food consumption, social influence on peers and media, lack of time among staff, and largely passive parent participation. Conclusions: Nutrition education was perceived as inadequate on its own to support sustainable behaviour related dietary change. Factors that may influence whether nutrition messages can be translated into practice were identified by the study participants and include the school food environment (canteen), the development of the curriculum, policy integration, and shift in parental participation from passive learning to active collaboration.

## 1. Introduction

Adolescence (10 to 19 years) represents a critical developmental stage characterized by rapid biological, psychological, and social and behavioural changes [1,2]. Globally, adolescents constitute nearly one-fifth and in Pakistan approximately 22% of the total demographic [3]. Behaviours are adaptive during this formative period, and often persist into adulthood. This age group is increasingly adopting unhealthy lifestyles, such as poor dietary habits, physical inactivity, and substance use, which contribute to the growing risk of non-communicable diseases (NCDs) later in life [4,5,6]. In Pakistan, the need for targeted, age-appropriate health interventions is clear, where NCDs are responsible for most of all age-standardized deaths [7].

**Figure 1:**
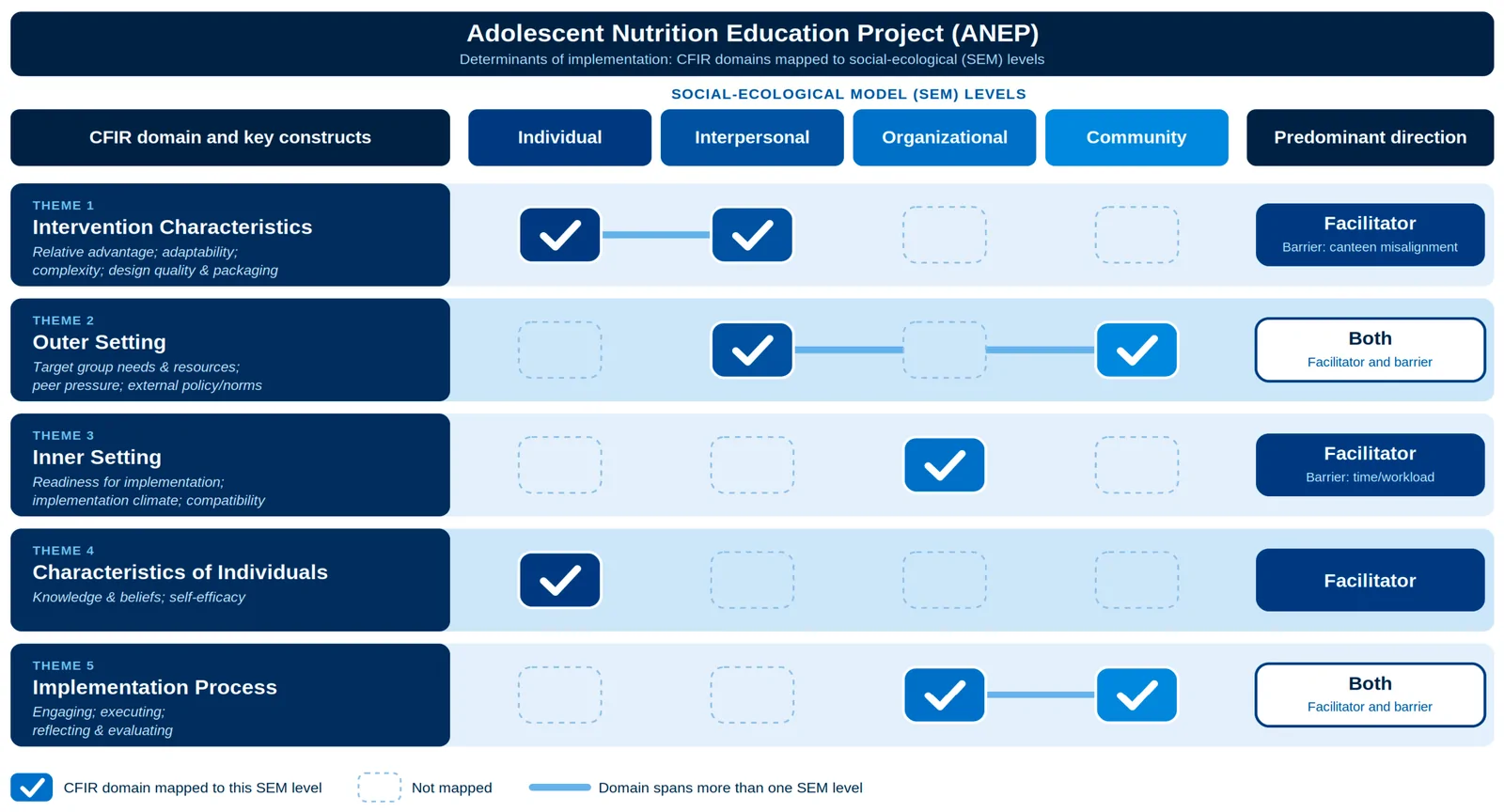
Conceptual diagram of the relationship between the CFIR domains and SEM levels in the implementation of ANEP. Note: CFIR domains were adapted from Damschroder et al. [15], and SEM levels were adapted from McLeroy et al. [16].

Despite the increasing burden of nutrition-related diseases in public health, prioritizing adolescent health in national nutrition policies remains insufficient. National surveys in recent years have highlighted the increasing rates of overweight and obesity along with undernutrition. However, few interventions have focused on adolescents. Existing initiatives are fragmented, with inadequate theoretical evidence and evaluation, creating a significant gap in knowledge on effective strategies that can influence adolescent behaviours and dietary habits in a resource-constrained setting [2,8,9,10]. Educational institutions offer an effective platform for promoting health during the formative phase of life [1,11,12,13]. This study evaluated a school-based nutrition education intervention, which is contextualized for Pakistani adolescents. The intervention was designed to transfer knowledge and to engage parents, peers and teachers, addressing broader ecological factors that influence dietary behaviours [14]. The current evaluation is grounded in the Consolidated Framework for Implementation Research (CFIR) and the Social Ecological Model (SEM). SEM conceptualizes health behaviours as shaped by interactions across individual, interpersonal, institutional, and societal levels. The CFIR provides a comprehensive scientific implementation framework that helps systematically identify factors influencing the adoption, implementation, and sustainability of interventions across five domains: intervention characteristics, inner setting, outer setting, characteristics of individuals, and implementation processes [15]. This allows for a multilevel exploration of implementation processes and outcomes (See Figure 1).

## 2. Materials and Methods

### 2.1. Study Aim

The aim was to identify facilitators and barriers to effective implementation of school-based nutrition education by applying the Consolidated Framework for Implementation Research (CFIR) and interpreting findings across levels of the Social Ecological Model (SEM).

### 2.2. Study Design and Theoretical Framework

A qualitative descriptive study design was employed, guided by CFIR. The approach was particularly appropriate for exploring participant perspectives through a structured lens to examine five key domains, i.e., intervention characteristics, inner setting, outer setting, characteristics of individuals and implementation process, in a naturalistic school setting. Additionally, the SEM was used as a sensitizing framework to explore the multlevel influences on adolescents’ dietary behaviour, spanning individual, interpersonal, organisational, and community domains.

### 2.3. Study Participant Selection

To capture a broader perspective across all the stakeholders, individuals who were directly involved in or could influence the intervention were enrolled in the study through purposive sampling. The Adolescent Nutrition Education Plan (ANEP) intervention was conducted on students from grade 6 to 8 in a private school. They were given six biweekly nutrition education sessions with the support of class teachers and the school coordinator [14]. The students received nutrition education through presentations, discussions and role-play. These sessions were conducted once every two weeks and delivered over twelve weeks (03 months). The content of sessions included holistic lifestyle (six pillars), MyPlate and dietary diversity, nutrition and disease prevention, sugary and processed foods and label reading, breakfast and mindful eating, and benefits of fruits, vegetables and hydration. The sessions were co-facilitated by class teachers and a certified nutritionist and were reinforced through a WhatsApp group and parent engagement sessions. Class teachers with science or health education backgrounds were selected for their familiarity with health-related content and daily contact with students. They received two-day training from the nutritionist, who also supervised delivery. For study participant selection, students who fully participated in the intervention and were willing to share their experience were included in the study. Two focus group discussions (FGDs) with eight students in each group were conducted. This group formation was considered as a suitable size for encouraging discussion along with allowing individual contributions, as supported by Krueger & Casey [17]. To explore the influence of age and development stage on shaping adolescents’ understanding of and responses to nutrition education, students from multiple grades were selected.

Teachers who facilitated the implementation of the intervention and had interacted with participating students were included in the study. This provided the opportunity to explore insights of the integration of the intervention within routine classroom practices. Four in-depth interviews were conducted with one section head who was involved in adjusting the class schedules to accommodate nutritional sessions, one class teacher and two other teachers who were involved in conducting nutrition sessions. Two key informant interviews were also conducted with the school administrators.

Parents/guardians of the students who completed the sessions were also included in the study sample, to explore interventional influence on dietary practices at their homes. A total of six parents, five mothers and one father, participated in individual interviews, offering perspectives on any behavioural or food-related changes observed in their children. This distribution reflects Pakistan’s cultural context, where usually mothers are primary decision-makers of household food preparation and were more available to participate in school-based sessions. Including multiple stakeholder groups enabled triangulation of perspectives and provided a more comprehensive understanding of the intervention’s influence across school and household contexts. Table 1 presents the summary of the participants characteristics. All individuals approached for study participation agreed for voluntary participation, and no participant withdrew after providing the consent.

### 2.4. Data Collection Procedure

Interviews and FGDs were conducted eight to ten weeks following the completion of the intervention to allow for reflection on the intervention’s impact. A semi-structured, interview guide was developed to explore participants’ experiences with the intervention, perceived facilitators and barriers to its implementation, and any observed behavioural or environmental changes following the programme (Supplementary File S1). The guide was informed by constructs from CFIR [15] and SEM [16]. Questions explored perceptions of the intervention content, feasibility, acceptability, contextual enablers and barriers, engagement strategies, and sustainability potential to ensure that questions captured influences operating at individual, interpersonal, and institutional levels as well as factors affecting implementation processes. There was a separate set of questions for each group of stakeholders. The guide was reviewed by experts in public health and qualitative research to ensure clarity, relevance, and content validity. It was piloted with a small subset of similar participants (n = 3). Minor modifications were made based on feedback to improve clarity, flow, and appropriateness of language, and were refined prior to data collection.

Separate data collection methods were used for different stakeholder groups to encourage open discussion and obtain both collective and individual reflections on the intervention. Students’ FGDs offered an interactive environment, where the participants were able to share experiences, compare perspectives, and reflect on the influence these nutrition sessions made on their knowledge and routine dietary choices. The interviews from other stakeholders provided the opportunity to capture a broader perspective on the intervention. The interview sessions lasted for 30 to 60 min and were audio-recorded with permission.

Two trained research associates holding Master’s degrees in Public Health and who had experience in qualitative interviewing and adolescent health research were selected as data collectors. They were not involved in delivering the ANEP intervention and had no prior contact with the school. Prior to study commencement they were given additional training on the study objectives, ethical conduct, and the application of the CFIR-based interview guide. To ensure consistency and adherence to study protocols, keen supervision was provided by the principal investigator during the data collection phase.

### 2.5. Data Management and Analysis

The COREQ (Consolidated Criteria for Reporting Qualitative Research) was used for reporting of this qualitative study [18]. Interviews and FGDs were mostly recorded in English. Occasional responses given in Urdu were transcribed verbatim and translated into English. The translated excerpts were checked against the audio recordings by two bilingual team members to ensure that the meaning of the perspective shared was preserved. The risk of loss of conceptual equivalence was considered minimal as the translated material was small. For maintaining data confidentiality, all transcripts were anonymized. Based on six-phase method outlined by Braun and Clarke [19], a thematic analysis was conducted.

Data saturation was assessed simultaneously with data collection and analysis. After each key interview or FGD, the two coders reviewed the transcript against the evolving codebook to identify new codes or perspectives. Data collection was considered saturated when additional data yielded no new codes or relevant information to the frameworks (CFIR and SEM) used in the study for analysis. The decision on saturation was reached by consensus between the coders.

For data familiarization, transcripts were read multiple times by the researchers. Using a combination of deductive codes informed by the CFIR, along with inductive codes that emerged from the data, initial coding was done. The codes were then organized into broader themes that reflected patterns of the ANEP implementation and experiences. Each theme was assigned the SEM level at which the determinant operated and where change would be required. For further refinement of themes for coherence and distinction, they were reviewed through comparison across participating groups. Lastly, mapped on CFIR domains and SEM levels, themes were clearly defined and explained with participants’ quotes; this provided a structured interpretation of the findings. Data was managed manually using Nvivo (Version 15.5.0, Lumivero, Denver, CO, USA); two independent researchers performed initial coding and developed a codebook collaboratively. To enhance analytical rigor, coding discrepancies were resolved through consensus meetings. Frameworks proposed by Naeem et al. and Nowell et al. were applied throughout the study to improve the quality of the qualitative findings [20,21]. Use of a structured interview guide based on CFIR and SEM and the principal investigator’s supervision during data collection enhanced the credibility of the study.

Maintaining an audit trail, writing reflexive journals, and engaging multiple researchers in coding and theme development strengthened the reliability and confirmability of the study. Triangulation was done through collection of views from students, teachers, parents, and school administration and through independent coding of two researchers. The potential transferability was addressed through the provision of detailed context for the setting and the participants involved, allowing the reader to judge the applicability of the results In other educational settings. The principal investigator supervised data collection and was involved in the broader intervention study. To manage the potential influence of PI, on decisions about data collection and interpretation, a semi-structured guide was provided, and reflective notes were maintained during the collection and analysis process. Two researchers independently coded the transcripts, and differences in interpretation were discussed until consensus were reached.

### 2.6. Ethical Considerations

This study commencement was carried out in alignment with the ethical principles of the Declaration of Helsinki. The Institutional Review Board of the Health Services Academy, Islamabad, Pakistan, provided the ethical approval for the study (Reference number: IRB No. 00011/HSA/PhD-368 2022). Permission to conduct the study was obtained from the participating school’s administration. Prior to data collection, written informed consent was obtained from all the participants. For adolescent participants, written informed consent was obtained from their parents/legal guardians, whereas verbal assent was obtained from the adolescents themselves. Participants were assured of the voluntary nature of their involvement and informed that they could withdraw from the study at any time without any consequences. All information collected was kept strictly confidential and used solely for research purposes. Data were anonymized before analysis to ensure participant privacy and minimize bias.

## 3. Results

The themes derived from the data collected are aligned with the domains of the CFIR framework. Perspectives of the participants, including students, teachers, parents, and school administrators, are contrasted under each theme in order to identify similarities and differences in their experiences of implementation of the nutrition education plan in schools.

### 3.1. Theme 1: Intervention Characteristics

#### 3.1.1. Subtheme: Perceived Value and Relevance of the Intervention

Stakeholders largely agreed that the intervention was timely and addressed a critical gap in school-based nutrition education. Parents, teachers, and administrators recognised that adolescent dietary habits had been long overlooked in formal curricula, despite their growing impact on health and academic outcomes. A parent stated that “I felt good and positive, as I believe that health education is important for our children,” reflecting both emotional and practical support. Likewise, another parent reflected on importance of this intervention by saying, “Now they talk about healthy eating while at home, before this they didn’t even care.”

The contextualization of the content was supported by the teachers. One of them shared her experience by stating, “We see students eating unhealthy on daily basis, but finally we were able to address it through these tools”. School administrators found the programme relevant, as it aligned with broader institutional goals. The principal said, “With good health, a child learns better, this initiative supports our long-term vision.”

Students described the sessions as unique and useful. A seventh-grade student expressed that “We were never taught about healthy eating like this before, it has brought awareness to us now.”

While most participants responded positively towards the intervention, a few from the teaching faculty initially showed their concern on the adjustment of this programme within academic schedules. They feared that it would increase their workload, and worried about managing along with the other academic priorities. One of them stated that “It is a useful concept, but fitting it into our tightly scheduled timetable was quite tough.” However, these concerns were later diminished after training, and even reluctant teachers appreciated the practicality of the intervention. Similarly, a few parents also questioned the sustainability of this programme once the research period ended, reflecting the uncertainty of institutional commitment to such interventions in the long-run.

Overall, the intervention was accepted by the majority and was considered as needed and relevant. However, concerns about its feasibility and sustainability were also raised regarding institutional support and commitment.

#### 3.1.2. Subtheme: Clarity and Accessibility of Content

The strength of implementation outside the classroom became evident in terms of the accessibility of the intervention. Educational information was strategically shared using the internet as a platform, where access to the key messages could be gained constantly. The use of WhatsApp as a tool by teachers was found to be useful for expanding the lessons learned in the classroom to the parents by sending messages about sessions and tips based on the weekly topic. As a teacher stated: “The WhatsApp group has helped us in keeping parents engaged, with what we were teaching at school. It was easy to share key points, send quick reminders, these simple actions helped in creating continuity between classroom learning and home practice.”

Parents acknowledged that the material shared was accessible, and simple in understanding. Also, as the material was shared on WhatsApp it was readily available when needed. A parent said that “When I receive messages on WhatsApp, I would know what my child was learning at school. It helped us reinforce the same learning at home.”

Students also expressed the need for sharing more Instagram content that is relevant to their age group, especially through short reels focusing on holistic approaches towards acquiring an improved lifestyle, including information about balanced meals, hydration, sleep, physical activity, and critical insights into habit transformations. These short clips were described as visually more appealing, relevant and customized to their age group. A student shared that “I found Instagram reels relevant to real-life habits, it provided practical advice on reducing junk food, physical activity, and sleep. Also, we could watch, save and share them anytime even after the study has ended.”

Another student appreciated reinforcement through repetition, “What we were taught in class was, also shared on our Insta accounts, this repetition of same information helped learn them well, and it stayed in our minds for long. The short reels give quick messages and were easy to remember and helped us consciously change our habits.”

The digital platform proved to be a useful yet complex part of the intervention. Visually appealing material and online discussion proved popular, especially amongst students and tech-savvy parents. The teachers noted that the digital interventions such as discussion, recipe exchange, and consultation made learning spill over from classrooms to homes. A father revealed that “I discussed my overweight problem, and got some useful practical advice for my weight loss. I followed it for a while, but couldn’t continue as my office routine is very tough.” Similarly, a mother shared, “I took advice for my autistic child, which was useful. I appreciate the support provided on the forum.”

Participants described the digital component as a useful source of reinforcement. However, the teachers reported that a few parents found regular digital messages intrusive or bothersome, and some even opted out of the communication group. Therefore, the study participants’ perception revealed how it is essential to consider digital fatigue when trying to encourage more parental engagement.

In general, the use of digital media proved to be an important addition to the intervention, providing additional support to the parents and emphasizing the key aspects of the programme. Nevertheless, the success of the process hinged on the need to stay relevant, show respect for the user’s comfort level, and promote digital accessibility to all participants.

Overall, the research findings point out that the technology was not only an important tool used to disseminate information but a structured reinforcement technique that helped to improve message retention, align parents, and ensure peer-to-peer message diffusion.

#### 3.1.3. Subtheme: Adaptability and Contextualization Within the School Environment

Contextualization was stressed by stakeholders as being critical to increasing relevance and sustainability of the intervention. It was observed that teachers recommended that the intervention be incorporated into the science curriculum. As one teacher pointed out, “It can easily fit into existing subjects.” For their part, parents stressed the importance of linking the intervention content with the reality of households, especially through incorporating local food choices. As one parent stated, “The nutrition sessions were relevant and practical for our daily life, as they advised about local food choices and considered cultural aspect too.”

There was clear appreciation from the students for the application of the activity-based learning strategies, such as group work and demonstration, which allowed them to see the relevance and applicability of the lessons taught. Notably, the students appreciated the fact that the materials used in the intervention were carefully crafted to reflect local eating habits and cultures. A student said, “We enjoyed our active engagement through group activities because they were related to our real life. It was practical as well as enjoyable, like we designed a healthy birthday menu and discussed what to order when eating out, it made us think creatively and in the way we’ve never done before.”

None of the participants expressed any problem with clarity of the content presented. The major problem was related to the inconsistencies of the environment within the school setting. There is a discrepancy between the messages conveyed during the sessions and the food options available in the school canteen.

The teachers felt that such inconsistencies would not help with the reinforcement process. The parents were very vocal about the discrepancies, with one commenting that “I think with canteens offering chips and sugar drinks, teaching healthy food practices in classroom makes very little sense.”

With unhealthy food options readily available, some students also shared the peer pressure that affects their food choices. When discussed, a major consensus from all study groups was derived that the unhealthy food available at canteens is not aligned with the nutrition education given in classes.

Parental representation demanded the development of a healthy lunch or canteen policy, arguing that the environment is important since teenagers are vulnerable to the influence of their peers. Despite the divergence of opinions among the representatives in regard to what should be stressed—teachers stressing curriculum while parents stressed the importance of aligning the issue at home and in the food services area—the participants were unanimous that adaptability should not be confined to education but should include the school environment as well.

### 3.2. Theme 2: Outer Setting

#### 3.2.1. Subtheme: Home Food Environment and Economic Constraints

The importance of home as one of the determinants of dietary behaviour among adolescents was clearly pointed out by all groups of stakeholders. It is clear that the ecological limitations of a school intervention programme can be experienced if such a programme is conducted without considering other factors, such as what happens at home. As one of the teachers observed, “School can only teach, but it is home that decides what their child will eat.” This is a reflection of an understanding of the limitations of influence by institutions after returning to one’s own environment.

The students Identified limitations that hampered their ability to use their knowledge in practice. While there was an acknowledgement of more awareness and desire to change lifestyle choices, it was also clear that food availability at home influenced consumption behaviour. One of the students mentioned, “We want healthy food, but sometimes only convenient foods are available.” This perspective reflects the adolescents’ partial participation within family food systems, where mostly adults are the decision-making authority and have purchasing power.

On the other side, parents put forward the economic realities like inflated food costs and budget constraints as major challenges to consistent implementation of healthier dietary practices. As one parent said, “Healthy food options are costlier than the unhealthy choices, we do try buying healthy, but with limited budget it is not always possible.” This framing has changed the discourse from personal responsibility to affordability challenges, which reflects socio-economic determinants of eating behaviours.

The general agreement on the importance of the family background was made; however, responsibility varied between teachers, parents and students. The former were focused more on parental and household decision-making influence. The latter paid attention to economic and contextual barriers affecting choice. Students saw themselves as people being in the specific environment created by availability. Overall, one can see how dietary behaviour is determined by the complex family environment where just having knowledge is not enough for making changes.

#### 3.2.2. Subtheme: Peer and Media Influence

Food selection by adolescents was largely influenced by the peer pressure and influence of media, as identified by the teachers, students, and parents. The teachers always talked about the strong effect of the social media on their eating behaviour. One teacher said, “I often observe that students discuss viral snacks or drinks on TikTok and Instagram. Constant exposure to these contents makes the temptation real”. Another said that online trends increase the peer pressure, “To fit in their online circle, these young individuals urge for sharing fancy eating, which often promotes unhealthy culture.”

With confused feeling, the students themselves acknowledged this influence. One shared that “Influencers set different trends for generating content, it looks cool and fun. When I see everyone is trying it, I also feel tempted towards it though I know it is not healthy or good for our health.” Another student spoke on peer dynamics, saying, “I have seen that some of my friends upload pictures and videos of cooking or eating fast food. Just to stay active in my friend circle and attract more likes on my profile I also try and upload pictures as they do. But I don’t do it every time, sometimes I am able to control these temptations.” This indicates that even though media and peer pressure have considerable impact, teenagers still have some knowledge about making appropriate choices.

However, parents have largely expressed negative opinions, and have highlighted that media and social platforms have a significant role in promoting unhealthy practices. One of the parents expressed that “They carry their cell phones all the time. Ads and promotion of snacks, fast food, and sugary drinks are everywhere on these platforms without any regulation. With so much on their social media feeds, it gets hard for our children to resist and their cravings increases for such things.” Another parent said, “even if I give them healthy food, they won’t eat it and would ask for things they have seen online or what their friends are eating. I feel this negatively affects their habits.”

In general, there was no doubt about the strong impact of media and peer pressure on the food preferences of adolescents. However, there were conflicting opinions concerning the effects of such an impact. While students understood that although social media and peers can be the source of unhealthy eating habits, it does not mean that there is no chance to discover new healthy recipes or become interested in healthy eating. Meanwhile, parents viewed the impact of media and peers as purely negative.

#### 3.2.3. Subtheme: Cultural and Social Norms as Barriers and Enablers

The cultural and social norms belonged to the Outer Setting level, because they are factors that affect the adolescent eating habits from outside the school environment and from a larger socio-environmental perspective. The cultural and social norms functioned as both enabling and constraining factors, influencing the use of healthier practices.

On one hand, established culinary practices would sometimes hamper the application of nutrition-related knowledge. Some participants reported typical examples of eating patterns including regular consumption of fatty snacks, over-consumption of red meat, eating dinner late at night, and organizing feasts that feature food products rich in calories. All of these habits were not treated by the interviewees as exceptions but rather as something that is normal in terms of social culture. Additionally, gender roles have added another barrier to the realization of healthy dieting practices. Teenage girls could be engaged in food preparation but seldom made choices about ingredients or methods of cooking. As one parent reflected, “Traditional food choices make it harder to apply the lessons at our homes.” Such responses exhibit the significance of customs and hierarchical family structures in defining the impact of newly acquired nutritional knowledge.

On the other hand, certain cultural practices served as effective enablers. The mothers were generally found to be willing to try changing the way their food was prepared; for example, preparing food with whole wheat flour or lowering the amount of oil used during cooking when given proper directions. In addition, many cultural foods were found to contain vegetables such as onions, tomatoes, garlic, and ginger that were included in stews or curries, and the culture of combining meat with vegetables became an accepted basis for well-balanced food. Instead of being a drastic change from the culture, healthy modifications could simply become an improvement on the present food habits.

Generally, while some cultural practices were in opposition to the nutritional guidelines, some others supported them. It can be seen that behavioural change is easier when the intervention considers the social reality and builds on the strengths that are embedded within the culture. It is essential for any intervention to recognize the cultural practices not just as barriers but also as strengths.

### 3.3. Theme 3: Inner Setting

#### 3.3.1. Subtheme: School Readiness for Implementation

The preparedness of the schools for the implementation of the nutritional education was found to be a critical variable in determining the success of the intervention. Most teachers valued the support of the school administration as they felt that it made the job easier. As one teacher stated, “Though time adjustment was a difficult task, but we were positive due to administrative support.” This reflects that despite institutional support, integration of an additional session was a challenge, and it concerned the teaching faculty. Another teacher reported that adjusting new activities alongside routine academic responsibilities is difficult and requires careful planning. Teachers felt the stress, as they were enthusiastic about the programme, but practical workload constraints were holding them back.

While appreciating the dedicated time and attention, students expressed positive experiences about the intervention. One student stated, “We enjoyed the special sessions, they gave us a break from our academic routine and also were useful”. These responses reflect that structured approaches for learning healthy habits are both enjoyable and productive. This excitement suggests that when a well-organized support is provided by the schools, students become receptive and are motivated for active participation.

The parents’ view of the necessity of institutional dedication and sustainability of the programme is clear from the following statement: “The school should include this in their routine timetables.” The parents were stressing the need to have the programme incorporated as a long-term part of the school curriculum, rather than a short-term endeavour. The attention paid by parents to the issue of sustainability clearly indicates their belief that it is the responsibility of the institution to provide an environment conducive to healthy habits.

In general, there is consensus that school can play a very positive role in nutrition education. Nevertheless, there are differences in views, which depend on whether the discussion is held with teachers or parents.

#### 3.3.2. Subtheme: Implementation Climate

Implementation environment at the schools was another factor that greatly influenced the success of the nutrition education initiative. Teachers felt that student interest helped them in becoming motivated to conduct classes. One teacher said, “Students showed great interest, that motivated us too,” indicating how the visible interest on the part of the students helped create an environment that motivated the teachers to put in efforts. Some other teachers also felt that watching the students become involved in the activity made them more creative and motivated, though there was a need for some policy on the part of the school.

From the students’ perspective, interactive learning became another factor that made the sessions interesting. One student said, “We found the interactive classes more appealing than typical lectures.” These responses reflect that the hands-on activities, demonstrations and discussion made the learning experience more enjoyable and impactful.

Parents observed that there were tangible but gradual changes in the behaviour of their children regarding food consumption. As noted by one parent, “I have noticed that, my child’s food preferences and choices have changed for good.” This reflects that the interventional effects have already started influencing the eating habits of the students at home. Additionally, parents also highlighted the need for a more structured and sustainable implementation of the programme.

Overall, it is clear that there was a consensus on the beneficial effects of using interactive methods to increase engagement and improve the implementation climate. Differences arose from different expectations and support needs; teachers required structured policy support to embed the programme into the regular school routine, and parents expected sustainable and measurable results.

### 3.4. Theme 4: Characteristics of Individuals (Knowledge, Beliefs, Motivation)

#### 3.4.1. Subtheme: Improved Nutrition Knowledge and Critical Awareness

The adolescents’ nutrition awareness and knowledge after the intervention was perceived as improved by all stakeholder groups. Teachers commented that students had gained better food literacy skills, especially in terms of being able to read food labels. For instance, as one of the teachers stated, “The students are now able to read the food labels correctly. They have started to look for things like ingredients and calories.” Adding up to this another teacher said, “Students have started to question more critically, like they question about healthiness of products that are labelled as low fat or while looking for refreshing juices, they ask if the box contains real fruit juice or not.”

Students also described applying this knowledge in daily situations, like one of the students shared, “After learning about the harmful effects of sugar intake, I have stopped buying sugary drinks.” Likewise, parents also observed that their young children are now bringing new information into the household environment. One parent said, “When they see me using too much oil while cooking, they correct me.” Another shared their experience at grocery store, saying that “My child has started reading food labels when I take him along for buying monthly groceries and also explains to me what is written on the labels.”

This shows that, apart from improving knowledge among students, the intervention was also perceived as an important step in enhancing the diffusion of nutrition awareness within the family context. Although the participants were largely in agreement on the improvement in awareness, some parents questioned whether this would last in the absence of continuous reinforcement.

#### 3.4.2. Subtheme: Increased Motivation and Self-Efficacy for Healthy Choices

Participants noted the increasing confidence of the students in choosing and talking about healthier eating habits. Teachers indicated that the children have become more inclined to participate in discussions and talk about nutrition terms. For example, one teacher stated, “Now the students feel confident while talking about nutrition.” Another one mentioned, “I have seen students debating with their fellows about what is healthy and what is not.” This shows that students felt themselves capable to express and apply knowledge.

Students themselves described a greater sense of independence in their food decisions. One student explained, “While at canteen during my break, I carefully choose snacks that are healthier, and double check before buying” Another stated, “After learning the good eating habits, I do not buy junk food, though my friends buy them.” Parents acknowledged this confidence but highlighted the influence of external environments. As one parent commented, “They do try, but still temptation is stronger than their willingness to opt for healthier choices.” Another said, “While at home they confidently discuss healthier choices, but when they go outside, they often skip the healthier options.”

In general, there was a consensus among all parties that the intervention helped students gain better knowledge and motivation to make healthy food choices. On the other hand, the issue of sustainability raised differences in opinions. While teachers believed that the students’ interest is a good indicator of sustainable empowerment, parents were more sceptical about sustaining these practices outside the school setting.

### 3.5. Theme 5: Implementation Process (Planning, Engagement, Execution, Reflection)

Within the CFIR context the implementation process was explored through four constructs: planning, engaging, executing, and reflecting and evaluating, whereas sustainability and scale-up themes emerged inductively from the data and were included as an additional subtheme.

#### 3.5.1. Subtheme: Engagement of Stakeholders

Stakeholder involvement was found out to be another very important determinant of the success and sustainability of the intervention. Teachers pointed out that the involvement of parents, which has become much more active and structured, has led to a significantly higher impact of the intervention. As one of the teachers put it, “Parents’ involvement on the social media has been an important aspect of the whole process.” It seems that reinforcement at home was necessary to make sure that the learning was consolidated. In the words of another teacher, “Though we were informing the parents, still we lacked structured regular communication and feedback. If we had conducted a workshop in the start or engaged them in activities, it might have generated even better results.”

Students also recognised the significance of their parents’ participation and expressed enthusiasm when engagement went beyond the classroom. One student noted, “I enjoyed seeing my parents participating through assignments, I felt that I am doing this with my parents as a team.” Another shared, “It felt like a serious task, when my parents discussed the homework with me.” This showed that collaborative activities generate a sense of shared responsibility and facilitate reinforcement of learning, compensating for the gap between the school–home learning environment.

The participation of parents in the programme was seen positively by the majority, and the chances given were considered enough for them to get all the required information regarding teenage nutrition. The two sessions arranged by the school gave the parents knowledge of the important messages in the intervention, and the updates via social media kept them informed about the different activities and session messages. As mentioned by a parent, “We were informed, and the sessions made us understand what our children were being taught.” One more parent shared that the updates that were shared helped them discuss the topic with their kids.

Nonetheless, it is apparent that although communication was efficient, there were not many joint events whereby parents and students could engage in any activity together. As one of the parents observed, “We did receive the information, but there was no joint activities arranged where both the parents and children could participate.” Regardless of this, the parents were happy that they were well-informed and willing to participate even further in case future events would include any interactive element.

The importance of increased involvement from parents to support and extend the influence of the project was unanimously agreed upon by all stakeholders involved. The difference arose in terms of who had the responsibility and opportunities. Parents were viewed as passive participants by teachers, while the challenge faced by parents was mainly structural in nature. This highlights the necessity of having a carefully designed strategy to support cooperative efforts and partnership.

#### 3.5.2. Subtheme: Monitoring and Feedback

Monitoring and feedback systems were considered integral in measuring the success of the intervention, although experiences differed from one stakeholder group to another. Teachers found it helpful that there were evaluation tools built into the programme. As one teacher put it, “The quizzes and discussions helped us evaluate our progress. It was inspiring to be able to evaluate our progress and see that the students really grasped the concept.” Another teacher added that “Without assessment tools, it would have been hard for us to justify the time we took. Assessments helped us prove what progress we made.”

The evaluative element was likewise appreciated by the students. “We felt proud to know that our scores were improving,” one of them noted. And another one said: “When we see the difference, it feels like we have accomplished something.” This evidence implies that the students’ sense of achievement and motivation has been strengthened by the feedback received, turning assessment into an encouraging experience.

In contrast, parents reported limited visibility into the formal monitoring process. While they were aware that assessments were conducted, many felt insufficiently informed about detailed outcomes. As one parent noted, “We didn’t see detailed feedback. We were told there was improvement, but we didn’t get a full report.” Another commented, “If we had received more structured feedback, we could have supported our children better at home.” This perspective indicates that although evaluation tools were functioning within the school context, parents felt excluded from the formal feedback loop, reducing their ability to reinforce progress externally.

Altogether, the evaluation mechanisms turned out to be a useful tool and contributed to demonstrating improvement. However, there was disagreement among the parents as to whether the process of feedback is inclusive enough. Teachers and students perceive assessment as positive and encouraging, while parents see the need for more communication and information.

#### 3.5.3. Subtheme: Implementation Strengths and Gaps

The process of implementation of the intervention was highly regarded by all implementers, teachers, and school management as being properly planned, contextualized, and implemented effectively, considering the short time available and the poor infrastructure. The teachers liked the fact that everything was properly organized and communicated. One senior teacher noted, “We were provided with everything required; training, materials, and monitoring; and thus, implementation did not affect other subjects.” The teaching staff were found to be friendly, well prepared, and ready to meet the needs of the schools.

The implementation team also stressed the flexible nature of the programme, whereby modifications could be made based on feedback. A teacher said, “We adapted to the mood in the classroom; it was not very rigid, and that is what helped to retain the students’ interest.” This reflects the programme’s flexible planning and delivery.

Nevertheless, some issues were also found. It was suggested by teachers and parents alike that there should have been a wider scope of the programme with regard to involving parents more often through group meetings. Although the contact sessions, digital messaging, and WhatsApp discussions worked, many agreed that the workshops would have had more effect.

Notably, several key informants pointed out that nutrition does not stand alone. Teachers and parents emphasized the need to consider various aspects related to an individual’s overall way of life, including physical activity, proper sleeping habits, and stress management—all of which influence an individual’s eating habits and general health condition. As one of the teachers explained, “Students go to bed late, skip breakfast, and watch TV non-stop; without considering these, it is difficult to change their eating habits.” This was perceived as necessary for future interventions in order to influence the target audience holistically.

A constant recommendation was the incorporation of the programme into the operations of the school as an institution. Teachers reported dissatisfaction about the lack of coherence between the lessons being taught in the classroom and the products offered at the school canteen. “We teach our students not to drink beverages with sugar, but in the canteen, they are still sold.” said one of the teachers. The teachers agreed about this recommendation, adding that it is hard for people to change their behaviour if the environment does not change as well.

One of the challenges that was encountered was lack of consistent involvement of auxiliary staff and management of the schools after initial acceptance. Although the leadership at the top level was supportive to the idea, its success depended on the teachers’ enthusiasm. As one of the teachers remarked, “It would have been better if such nutrition education plans are made part of the school policy, instead of just a one-time project.”

In conclusion, the strengths in terms of the intervention include the way it was presented and implemented, as well as its flexibility. On the other hand, there is a need to address shortcomings such as lack of parental support and environmental support (in the form of canteens). This could improve the impact of the intervention programme.

#### 3.5.4. Subtheme: Sustainability and Scale-Up Potential

It was clear that all stakeholders, educators, and parents agreed on the enormous potential of sustainability and replicability of the intervention. It was stressed that this programme tackled a very crucial area in health promotion at schools that had been neglected, and it should not be just a one-time event. As one of the school administrators stated, “This is not where we stop, this needs to go on.”

Nutrition education within the formal classroom curriculum was especially emphasized by the teachers; they believed that integrating the teaching of nutrition education in subjects like science and literature would contribute to its implementation. Some people also stressed the importance of training staff periodically so that the approach can remain consistent through time. As one teacher put it, “There are new teachers who come each year, there should be some system of orientation.”

Policies that should be developed at the level of schools for having a healthy environment were another main recommendation made by the participants. This was considered crucial for having consistency between the educational curriculum and other aspects of the environment, such as regulation of the canteen and promotion of physical activities.

The third area that was brought out was parental involvement. Though the initial digital approach was welcomed by the participants, some of them mentioned the importance of implementing strategies to engage parents in the process. Such strategies include having workshops at certain intervals, peer group support programmes, and co-curricular activities for families. “This is supposed to be an integral part of every school’s routine programme,” said a parent.

Replication of the programme was also mentioned as an implementable strategy that can be considered possible once it is contextualized and supported by professionals. Overall, the intervention was deemed not just useful but critical for future adolescent health promotion. The success of its sustainability and scalability depends on its formal incorporation into curricula, policy backing, training, and parent–school collaborations. The following table provides an overview of themes and subthemes derived from CFIR and SEM constructs. Table 2 summarizes the themes and subthemes derived based on CFIR and SEM constructs.

## 4. Discussion

The current paper findings reflect that success of such nutrition education plans for school adolescents’ integration at different levels is of significant importance, including the intervention design, institutional readiness, individual capacity, and the broader environmental factors, including social and physical aspects. This finding is broadly reflected in the existing literature of implementation science on school nutrition programmes.

In the current paper, stakeholders have emphasized the relative benefit and adaptability of the nutrition education programme in schools, which is well supported by the evidence generated in a systematic review including studies conducted in low- and middle-income countries (LMICs) on barriers and facilitators to implementation of nutrition-related actions within school settings [22]. However, a significant implementation gap was highlighted between the classroom education and canteen services, which contradict each other. Internationally, researchers have indicated that nutrition education alone cannot translate into sustainable behaviours without supporting environments. For sustainable change, institutional food-environment policy plays an important role in making the nutrition education programmes successful. A narrative review from New Zealand has highlighted that the school food environment is often obesogenic rather than conducive to healthy food choices due to canteen profit models, lack of staff compliance, and higher availability of low-cost unhealthy food options [23]. Similarly, a qualitative study from Malaysia reported that inconsistent food availability significantly undermines programme credibility among students and parents [24].

The Outer Setting theme identified the dual effect of peer group and social media usage. This is corroborated by findings from a recent adolescent nutrition research paper, which reports the pervasive availability of non-core, branded food content across adolescents’ social media feeds and the normalizing effect of peer-shared food imagery on dietary aspiration [25]. The cultural aspect analysed in this study aligned with qualitative research outcomes conducted on Pakistani populations, where the shift in the dietary behaviours of people is shaped by family hierarchy, communal eating practices, and the role of food in social life, in addition to an individual’s knowledge or intention [26]. The participants of the current study viewed culture not only as an impediment to behaviour change; the cultural component in their lives provided a platform for reinforcing their existing eating habits.

Results obtained in the Inner Setting domain, showing administrative support is mitigated by time and workload restrictions, are in line with the wider implementation literature, which indicates that availability of staff time is one of the most common barriers to nutrition-related action, despite having organisational commitment [22,27]. It implies that the sustainability of the ANEP project will be dependent on gaining additional staff time rather than further conceptual endorsement from the school administration, which seems to exist.

The domain Characteristics of Individuals was marked by steady improvement in nutrition knowledge, nutrition-label reading skills, and self-confidence. This is consistent with studies that show that reinforced multiple channels of delivery approaches such as classroom teaching combined with repeated messages through digital means have been considered feasible and acceptable ways of consolidating adolescent health knowledge in a systematic review of text message interventions to adolescent weight-related behaviours [28]. Parental concerns about the longevity of these improvements outside the school settings have turned out to be a major problem. For interventions like the one assessed in this research, it is easier to gain knowledge and motivation than a behavioural shift in the absence of reinforcement and supervision plans.

Findings from this qualitative study provide valuable information on the complex dynamics affecting the nutritional status of adolescents in schools. Awareness versus action was one issue that emerged clearly through all stakeholder interviews. This finding concurs with the existing literature [22]. Parents’ interviews showed fear and reluctance, despite recognizing that children needed to eat well; socio-economic factors along with a lack of supervision from schools prevented them from making sure their children ate properly. Teachers and school authorities recognised the significance of nutritional education but were unable to implement this due to lack of institutional support and training. Such results have been observed by previous researchers too [24,29,30]. These results highlight the importance of an intervention that is systematic, contextual, and education-based rather than just informational because it will target the socio-environmental determinants of behaviour, which have been documented to influence food behaviours [31,32]. The identified disparity between policy intention and reality calls for change on a systemic level that includes the inclusion of nutrition in the curriculum, teacher training, and approaches for communicating with parents and adolescents as advocated in other studies [10,33,34,35].

As a whole, the five topics address the individual (knowledge, self-efficacy), interpersonal (peer influence, food practices at home), institutional (school readiness, canteen setting), and community (cultural setting, digital/media environment) levels of the SEM, which recognizes the underlying assumption of the intervention, that the eating behaviour of adolescents cannot be discussed only from the perspective of what happens in class. This is also consistent with one known shortcoming of CFIR as such, which is the fact that while CFIR offers a detailed categorization of implementation determinants, as pointed out in a systematic review on the application of CFIR in LMIC contexts, the framework does not imply relations between CFIR constructs and distinctions between various levels of ecology [27]. Mapping CFIR constructs to SEM levels explicitly, as done in Table 2, could be an approach to solving this problem.

The trustworthiness of our findings was ensured through methodical rigor. Integration of the data across diverse stakeholder groups allowed for the validation of emergent themes. Reflexivity was sustained during the analysis process through the careful examination and recording of the researchers’ assumptions so as to reduce bias. Data was collected by independent researchers with no prior involvement in the previous interventional phase of the broader study, which minimized the risk of interviewer bias. Since other team members were involved at different points in the broader study, the PI and other team members discussed analysis, with respect to how its assumptions and association with the intervention might shape interpretation. Although there are several strengths in this research, there are some limitations that need to be noted. First, the research was undertaken in a private school within an urban district. In Pakistan’s education system, private educational institutes are financed through out-of-pocket fees; therefore, generally their students come from middle- to higher-income households. Likewise, the participating parents were comparatively highly educated, with all having at least graduate-level education; therefore, the study findings may not reflect the circumstances of families in public schools, or from lower socio-economic communities. Additionally, the canteen was run independent of school administration, by private vendors on a contract basis, rather than directly by the school administration. All these factors raise concerns of lack of transferability to other regions, especially those located in rural settings and with a distinct socio-cultural environment. Another limitation is related to potential selection bias, as only students and parents who had participated in the ANEP intervention and completed all sessions of the programme were included. This may have under-represented the perspective of those who dropped out or participated less in the interventional phase. Another aspect of the study was that since the data were collected post-intervention and were based on retrospective stakeholder perceptions, this study cannot therefore verify behaviour change or determine whether any changes were sustained over time. Prospective, longitudinal studies are need to establish this gap. Lastly, social desirability bias is possible, particularly in students who may have responded in a favourable manner, as the study was conducted in the school setting, and intervention was delivered by their own teachers. This risk of bias was minimized by involving independent data collectors who had not been part of the intervention. Moreover, study participants were also assured that their responses would not affect their relationship with the school.

Nevertheless, there is still great value in the insight provided because it will have practical implications for public health initiatives and education. Further research must look into the long-term effects of the school-based intervention and its capacity for scalability within different types of school settings. Through the use of the lived experiences of both adolescents and their parents, this study provides a very crucial framework to design effective interventions.

## 5. Conclusions

The qualitative analysis in this paper reveals that the successful implementation of a school-based nutrition education intervention for adolescents is not based on curriculum content only; it requires a multi-aspect approach, engaging school nutrition environment, policy implementation, and engagement of multi-stakeholders at different levels. By applying both CFIR and SEM as a set of tools that complement each other, the research concluded that positive outcomes in the form of increased nutrition knowledge, critical food literacy and self-efficacy among the adolescents were perceived consistently by all parties involved—the students, teachers, and parents. The process of communication was facilitated by an organized and multichannel approach that incorporated classroom education along with digital reinforcement; however, individual gains achieved through the use of the campaign were hindered consistently by outer- and inner-setting barriers. These include a conflicting food environment at school (especially unhealthy canteens). Though the aspect of institutional readiness existed theoretically, it was compromised in reality due to lack of adequate staff time and lack of policies that were embedded, while parent involvement was also not active because there were no structured engagement processes involved. It is thus evident from the above discussion that nutritional education alone cannot lead to behaviour change in the long run; for such sustainability, it is important to have formal curriculum integration, food environment policy integration (like regulating canteen services to ensure the availability of health food options), and a structured parent engagement process (encouraging active participation), among other factors. Further research should be conducted to check the sustainability of the effects of the above interventions as well as their scalability to different school settings.

## Figures and Tables

**Table 1:** Study participant characteristics (n = 28).

Stakeholder Group	Data Collection Method	n	Composition
Students	Focus Group Discussion (2 groups)	16	Grades 6–8; mixed gender; regular ANEP attendees
Teachers	In-Depth Interview	4	1 section head; 1 class teacher; 2 session-delivering teachers
School Administrators	Key Informant Interview	2	Administrators overseeing school-level implementation
Parents	In-Depth Interview	6	5 mothers, 1 father

Note: Each student FGD comprised four males and four females (8 males and 8 females in total). All four teachers taught science or health education subjects. Parents’ educational level varied, but all had at least a graduate-level education.

**Table 2:** CFIR-SEM construct to theme summary mapping.

CFIR Themes	Subthemes	Key Constructs Coded	Corresponding SEM Level(s)	Predominant Direction
Intervention Characteristics	Perceived Value and Relevance of the Intervention Clarity and Accessibility of content Adaptability and contextualization within the school environment	Relative advantage; adaptability; complexity; design quality & packaging	Individual; interpersonal	Facilitator: relevance, local content, digital reinforcement Barrier: canteen misalignment, digital fatigue
Outer Setting	Home food environment and economic constraints Peer and media influence Cultural and social norms as barriers and enablers	Target group needs & resources; peer pressure; external policy/norms	Interpersonal; community	Facilitators: culturally embedded health foods, mothers’ willingness to modify cooking Barriers: Home food availability, food cost, peer and media influence, traditional norms and gender roles.
Inner Setting	School readiness for implementation Implementation climate	Readiness for implementation; implementation climate; compatibility	Institutional/organisational	Facilitator: administrative support; student interest; interactive methods Barrier: teacher workload; no school policy
Characteristics of Individuals	Improved nutrition knowledge and critical awareness Increased motivation and self-efficacy for healthy choices	Knowledge & beliefs; self-efficacy	Individual	Facilitator: improved knowledge; confidence and self-efficacy Barrier: peer temptation outside school; doubts about lasting change
Implementation Process	Engagement of stakeholders Monitoring and feedback Implementation strengths and gaps	Planning; engaging; executing; reflecting & evaluating	Institutional; community	Facilitator: planning, training and materials; flexible delivery; quizzes and feedback Barriers: limited parent engagement and feedback; weak management involvement; not embedded in policy

## Data Availability

The data presented in this study are available on request from the corresponding author, due to the institutional policy of the first author.

## Supplementary Materials

The following supporting information can be downloaded at https://www.mdpi.com/article/10.3390/nu18213652/s1, Supplementary File S1: Semi-structured Interview and Focus Group Guide.

## Abbreviations

The following abbreviations are used in this manuscript:

CFIR	Consolidated Framework for Implementation Research
SEM	Social Ecological Model
NCDs	Non-communicable Diseases
FGD	Focus Group Discussion
ANEP	Adolescent Nutrition Education Plan
COREQ	Consolidated Criteria for Reporting Qualitative Research
LMICs	Low- and middle-income countries
