# Supplementary material for: Implementation Experiences of a School-Based Adolescent Nutrition Education Intervention: A Post-Intervention Qualitative Study Guided by Consolidated Framework for Implementation Research and the Social Ecological Model
Source: Nutrients. 2026 Nov 3;18(21):3652. doi: 10.3390/nu18213652 (PMC13766017; doi:10.3390/nu18213652)
Supplement: Supplementary file 1 [file nutrients-18-03652-s001.zip › nutrients-4625871-supplementary.pdf]

Supplementary File S1

Semi-structured Interview and Focus Group Guide

Study Title: *"Implementation of the Adolescent Nutrition Education Project (ANEP): a qualitative study guided by the Consolidated Framework for Implementation Research (CFIR) and the Social-Ecological Model (SEM)"*

PART A: General Background

- **Can you tell me about your role in the school?**
- **How were you involved in the nutrition education sessions or related activities?**
- **What were your first thoughts when you heard about the nutrition program?**

PART B: CFIR Domains and SEM level

1. Intervention Characteristics (SEM level: Individual, Interpersonal)

- **What did you think of the materials or activities?**
- **Were the messages clear and easy to understand?**
- **What parts did you find most useful or effective?**
- **What should be changed or improved?**

---

2. Outer Setting (SEM level: interpersonal, community)

- **How did people outside of the school respond?**
- **Were there social or cultural beliefs that influenced reception?**
- **Were there expectations from the community or school administration?**

---

3. Inner Setting (SEM level: Organizational)

- **Was the school environment supportive?**
- **Did the school provide enough time, space, and resources?**
- **Did teachers or staff communicate well about the program?**

---

4. Characteristics of Individuals (SEM level: Individual)

- **How motivated were you or your child to participate?**
- **What changes, if any, have you noticed in knowledge, attitudes, or behaviors?**
- **How confident did you feel in applying what was taught, and why?**
- **What personal challenges you have faced, if any?**

---

5. Implementation Process (SEM level: Organizational, community)

- **How well was the program planned and delivered?**
- **Were you given a chance to give feedback or ask questions?**
- **Who helped make the program work best?**
- **Suggestions for improving the program?**

---

6. Sustainability Potential (SEM level: organizational, community)

- **Should this program continue? Why or why not?**
- **What would be needed to continue without external support?**

- **What role could students, teachers, or parents play?**
- **School policies or systems that could help or hinder?**
- **Advice for other schools?**

PART C: Closing Questions

- **What lasting changes have you seen?**

- **Would you recommend this kind of program? Why or why not?**
- **Anything else to share?**
